# Vascular disease and apathy symptoms in the very old: A cross‐sectional and longitudinal meta‐analysis of individual participant data

**DOI:** 10.1002/gps.5831

**Published:** 2022-10-31

**Authors:** Veerle M. G. T. H. van der Klei, Rosalinde K. E. Poortvliet, Jonathan M. K. Bogaerts, Jeanet W. Blom, Simon P. Mooijaart, Ruth Teh, Marama Muru‐Lanning, Leah Palapar, Andrew Kingston, Louise Robinson, Ngaire Kerse, Jacobijn Gussekloo

**Affiliations:** ^1^ Department of Gerontology and Geriatrics Leiden University Medical Center Leiden The Netherlands; ^2^ Department of Public Health and Primary Care Leiden University Medical Center Leiden The Netherlands; ^3^ School of Population Health University of Auckland Auckland New Zealand; ^4^ James Henare Māori Research Centre University of Auckland Auckland New Zealand; ^5^ Population Health Sciences Institute Faculty of Medical Sciences Newcastle University Campus for Ageing and Vitality Newcastle Upon Tyne UK

**Keywords:** atherosclerosis, meta‐analysis, older people, prospective, vascular apathy

## Abstract

**Objectives:**

Previous findings suggest a vascular foundation underlying apathy, but transdiagnostic and prospective evidence on vascular apathy is scarce. This study examines the association between vascular disease and the presence and development of apathy symptoms in the very old.

**Methods:**

Four cohorts of the *Towards Understanding Longitudinal International older People Studies* (TULIPS)‐consortium were included in a two‐staged, individual participant data meta‐analysis using generalized linear mixed models. Vascular disease was defined as a history of any clinical atherosclerotic pathology (angina pectoris, myocardial infarction, intermittent claudication, transient ischemic attack, stroke or related surgeries) and was related to apathy symptoms as repeatedly measured by the Geriatric Depression Scale (GDS‐3A ≥2) over a maximum of 5 years.

**Results:**

Of all 1868 participants (median age 85 years old), 53.9% had vascular disease and 44.3% experienced apathy symptoms. Participants with vascular disease had a 76% higher risk of apathy symptoms at baseline (odds ratio (OR) 1.76, 95% confidence interval (CI) 1.32–2.35), irrespective of depressive symptoms and only partially explained by stroke. Conversely, there was no association of vascular disease with the occurrence of apathy symptoms longitudinally, both in those with apathy at baseline (OR 1.00, 95% CI 0.84–1.20) and without (OR 0.96, 95% CI 0.84–1.09).

**Conclusions:**

Vascular disease in the very old is associated with apathy symptoms cross‐sectionally, but not proven longitudinally, independent of depressive symptoms. These findings query a vascular cause underlying apathy symptoms. However, the consistency of our cross‐sectional findings in direction and magnitude across the TULIPS‐consortium do emphasize international relevance of the interplay of vascular factors and apathy in advanced age, which meaning needs further unravelling.

## Key points

- Apathy symptoms are a common burden in the very old population of which the complex pathophysiology is yet to be unraveled.
- Vascular disease might predispose older adults to developing apathy, but prospective evidence on a vascular origin for apathy in old age remains scarce.
- Vascular disease is consistently associated with increased risk of concurrent apathy symptoms, but there is no evidence for influence on future apathy symptoms in the very old.
- Optimizing cardiovascular disease (CVD) management to attempt to prevent apathy may not be warranted, at least in the very old.

## 1 INTRODUCTION

Reduced motivation, or apathy, is defined as a persistent lack of goal‐directed behaviour which impairs daily functioning.^1^, ^2^ Apathy symptoms do not merely occur with different medical conditions like dementia or depression,^3^, ^4^, ^5^, ^6^, ^7^ but are also common in the general population at old age. These symptoms affect community‐dwelling older adults in approximately 20%–40% and occur in the absence of depressive symptoms (i.e. isolated apathy) in 13.8% on average.^8^ Due to this widespread occurrence and little evidence about apathy prevention or recovery,^7^, ^9^, ^10^ there is a strong need for a transdiagnostic approach to unravel the complex pathophysiology underlying these symptoms.^9^

Vascular pathologies have been proposed to specifically precede apathy symptoms and not depressive symptoms, and to predispose older adults to developing apathy in old age: the so‐called vascular apathy hypothesis.^11^ An increasing body of literature has since emerged on the interplay between apathy and several proxies of cardiovascular disease (CVD), as comprehensively depicted in a recent systematic review.^12^ Various mechanisms could drive the vascular foundation of apathy, such as atherosclerosis or structural brain damage following stroke.^4^, ^9^, ^11^, ^13^ Conversely, apathy symptoms also seem to contribute to incident CVD,^14^ which could for example, be mediated by adverse health behaviour if motivation for health promotion declines.^15^ To our knowledge, only the latter perspective has been examined over time in a thorough meta‐analysis,^8^ whereas prospective evidence on the vascular apathy hypothesis remains scarce^12^ as well as replication of previous cross‐sectional findings in the very old population (i.e. those over 80 years of age),^11^ hampering further understanding about the direction and interpretability of these associations in old age.

Therefore, we aim to investigate the association between vascular disease and the presence as well as development of apathy symptoms in the very old, irrespective of depressive symptoms.

## 2 MATERIALS AND METHODS

### 2.1 Study design and participants

Our two‐stage meta‐analysis incorporated individual participant data (IPD) from the pre‐existing *Towards Understanding Longitudinal International older People Studies* (TULIPS)‐consortium.^16^, ^17^, ^18^, ^19^ This is a worldwide collection of prospective, population‐based studies on ageing in the very old, which was at the time established to increase power and to examine international variation in associations in old age. We included four of its cohorts from three different studies: (1) the Leiden 85‐plus Study from the Netherlands (2a) the Māori (indigenous population of New Zealand) and (2b) the non‐Māori parallel cohorts of the Life and Living in Advanced Age: a Cohort Study in New Zealand (LiLACS NZ) and (3) the Newcastle 85+ Study originating from the United Kingdom. In contrary to previous IPD meta‐analyses in the TULIPS‐consortium, the Tokyo Oldest Old Survey on Total Health from Japan had to be entirely excluded from this analysis due to unavailability of measurements on the outcome.

The Leiden 85‐plus Study is an observational study established from September 1997 to September 1999, which included 599 people out of the 705 eligible older adults that lived in the town of Leiden and turned 85 years old during the baseline period (i.e. a response rate of 87%).^11^, ^20^ LiLACS NZ was initiated between March 2010 and April 2011 and successfully recruited 421 Māori of 80–90 years old along with 516 non‐Māori of 85 years old residing in the Bay of Plenty and Rotorua area (56% and 59% respectively of all eligible older adults).^21^, ^22^ The Newcastle 85+ Study was set up in 2006 to 2007 by including 1040 85‐year‐old adults out of the sampling frame of 1470 people, which were born in 1921 and were registered with a participating general practice in Newcastle or the North Tyneside (i.e. a 71% response rate).^23^ All the above final samples were shown to be by and large representative of the several ageing source populations, as outlined in detail,^20^, ^21^, ^22^, ^23^ and within these samples individual participants were solely excluded from this study due to data unavailability on the exposure and/or outcome.

Repeated data collection was annual over a maximum of 5 years in the Leiden 85‐plus Study as well as LiLACS NZ, while follow‐up was clustered at 1.5, 3 and 5 years from baseline in the Newcastle 85+ Study. The individual study protocols were approved by Medical Ethical Committees at their respective sites and all very old or their proxies had granted either oral or written informed consent.

### 2.2 Vascular disease

Several manifestations of vascular disease have been assessed at baseline by combining different types of data collection per study (Supplementary Table 1). In the Leiden 85‐plus Study certified and trained research staff conducted standardized interviews with physicians.^11^ In LiLACS NZ such interviews were performed with participants and were seconded by primary health care and hospitalization record checks.^24^ In the Newcastle 85+ Study primary health care records were thoroughly examined.^23^ Electrocardiograms were recorded in all studies to objectify and to complement findings on previous myocardial infarction.^11^, ^23^, ^24^ Vascular disease (yes/no) was defined as a history of any clinical atherosclerotic pathology: angina pectoris, myocardial infarction, intermittent claudication, transient ischemic attack, stroke or any related coronary or peripheral artery surgery.

### 2.3 Apathy and depressive symptoms

All studies repeatedly administered the 15‐item Geriatric Depression Scale (GDS) to their participants,^25^ which is appropriate to assess symptoms over time.^26^ In line with other population‐based studies and prior factor analyses,^11^, ^13^, ^27^ a 3‐item apathy subdimension has been used to screen for apathy symptoms, which included the following questions: (1) Have you dropped many of your activities and interests? (2) Do you prefer to stay at home rather than to go out and do new things? (3) Do you feel full of energy? (GDS‐3A; range 0–3 points, with greater scores implying more apathy). Since scores ≥2 points have shown to be indicative of a clinical diagnosis of apathy, with a sensitivity of 69% and a specificity of 85%,^11^, ^28^ this cut‐off has been applied to dichotomize the presence of apathy symptoms. Likewise, the 12 remaining items of the GDS were summed up (i.e. those not reflecting apathy, the GDS‐12D; range 0–12 points, with greater scores indicating more depression) and a ≥2 point cut‐off was applied to reflect the presence of depressive symptoms.

As apathy is increasingly regarded as an independent syndrome across disease boundaries^2^, ^9^ and vascular apathy is preferably studied independently of depression,^12^ we classified apathy symptoms without these depressive symptoms as isolated apathy (concurrent GDS‐3A ≥ 2 and GDS‐12D < 2).^8^

Because the 15‐item GDS is self‐reported and its validity is decreased in older adults with dementia,^29^ the GDS was either not administered to participants with a Mini‐Mental State Examination (MMSE) score <19 points^11^, ^30^ or we excluded their GDS‐scores to align data availability between the cohorts (Supplementary Table 1). Exclusion of these GDS‐scores was carried out by assigning all GDS‐measurements in which the concurrent MMSE‐score was below 19 points to missing.

### 2.4 Other measures

Data on demographic variables such as age, gender, educational attainment (up to and including secondary school vs. higher education) and living situation (institutionalized or not) was gathered during the baseline interviews. LiLACS NZ participants also reported their ethnicity, whether they identified themselves as Māori, which was prioritized over other ethnicities if more than one was listed.^21^ According to overall data availability and previously applied conditions^11^ participants were considered to have a chronic disease when any of the following diseases was present: arthritis, cancer, diabetes mellitus, chronic lung disease or Parkinson's disease. This was evaluated by combining reported histories, related medication use and blood glucose levels, which are specified in the supplement (Supplementary Table 1).

### 2.5 Statistical analysis

A two‐stage IPD approach was adopted to acknowledge the heterogeneity between the cohorts and to honour the preferences and practices in NZ Māori research,^31^ which means the analyses were initially performed per cohort and their distinct results were afterwards pooled for meta‐analysis.

#### 2.5.1 Cohort‐level analyses

Generalized linear mixed models were applied as these mixed models optimize the use of available data during follow‐up and account for dependency of repeated measures per person.^32^ Two models were assessed in accordance with existing literature^11^, ^13^: (1) adjusted for age (only in Māori), sex and depressive symptoms (GDS‐12D ≥ 2, as a time‐varying covariate) and employing a random intercept, and (2) additionally adjusted for educational attainment and chronic disease. Besides the aforementioned exclusion of total GDS‐scores based on MMSE‐scores, no adjustment was made for cognitive status as it was thought to possibly be part of the causal pathway of interest. The selection of the final model is described in more detail in Supplementary Table 2. The cross‐sectional (main effect of vascular disease) as well as the longitudinal estimates (interaction term: vascular disease * time since baseline) were generated by use of the Lme4 package in R (version 4.0.2).^33^, ^34^ These estimates were respectively presented as baseline and annual odds ratio (OR) of apathy symptoms (GDS‐3A ≥ 2) with a 95% confidence interval (CI) (95% CI). The latter represents the additional OR with every year increase in time since baseline when having vascular disease and was stratified by baseline apathy status. All analyses were repeated while excluding participants with a history of stroke as a proxy for subsequent structural brain damage.^11^, ^13^

#### 2.5.2 Pooled analyses

The individual ORs were thereafter pooled by random‐effects models with inverse‐variance weighting and summarized in forest plots by using Review Manager 5.4.^35^ Consistency between the estimates was defined as a statistical heterogeneity (I^2^) below 40%.^36^

#### 2.5.3 Sensitivity analyses

To examine whether our findings were dependent on the cut‐offs that were applied to define apathy and depressive symptoms, we conducted sensitivity analyses by use of the ordinal package.^37^ We entered the continuous instead of the dichotomized GDS‐3A and GDS‐12D scores in ordinal logistic regression models, which were apart from that equal to the main analyses.

## 3 RESULTS

### 3.1 Demographic and clinical characteristics

Out of the 2576 participants of the TULIPS‐consortium we excluded 706 older adults due to data unavailability (27.4% in total; exclusion on MMSE accounted for 1.5%). This meta‐analysis therefore consisted of 1868 participants across the four cohorts and their baseline characteristics are shown in Table 1. The median age was 82 years old in the NZ Māori population and 85 years in the other cohorts. The majority of participants was female (60.1%), community‐dwelling (i.e. 5.6% was institutionalized) and maximally attained primary or secondary school (83.8%). Most of them were diagnosed with one or more chronic disease (77.7%) and their median MMSE‐score was 28 out of 30 (interquartile range (IQR) 26–29). Vascular disease was present in 53.9% of participants, explained by at least a stroke in 11.6% of all participants. Apathy symptoms were experienced by 44.3% of older adults, depressive symptoms by 34.3% and isolated apathy occurred in 22.8% of participants.

**TABLE 1:** Baseline characteristics of study participants and median follow‐up time

Cohort	Leiden 85‐plus study	LiLACS NZ		Newcastle 85+ study	Overall
		Māori	Non‐Māori		
*N* =	493	243	376	756	1868
Study population (starting year)	Cohort of 85‐year old inhabitants of Leiden, the Netherlands (1997)	Cohort of 80–90 year old Māori, indigenous to New Zealand (2010)	Cohort of 85‐year old non‐Māori inhabitants of New Zealand (2010)	Cohort of 85‐year old inhabitants of Newcastle upon Tyne, the UK (2006)	
Demographics					
Age, median [IQR], years	85 [85, 85]	82 [81, 84]	85 [85, 85]	85 [85, 86]	85 [85, 85]
Female, *n* (%)	312 (63.3)	148 (60.9)	199 (52.9)	463 (61.2)	1122 (60.1)
≤ Secondary school, *n* (%)	421 (85.9)	204 (84.3)	268 (72.4)	663 (87.8)	1556 (83.8)
Institutionalized, *n* (%)^a^	56 (12.1)	3 (1.3)	13 (3.5)	31 (4.1)	103 (5.6)
Clinical characteristics					
Chronic disease, *n* (%)^b^	296 (61.7)	187 (77.6)	322 (86.1)	614 (82.3)	1419 (77.1)
MMSE‐score, median [IQR]	27 [24, 28]	28 [26, 29]	28 [27, 29]	28 [26, 29]	28 [26, 29]
Depressive symptoms, *n* (%)^c^	144 (28.8)	73 (30.0)	86 (22.9)	337 (44.6)	640 (34.3)
Apathy, *n* (%)					
Apathy symptoms^d^	136 (27.6)	94 (38.7)	151 (40.2)	447 (59.1)	828 (44.3)
Isolated apathy^e^	68 (13.8)	56 (23.0)	99 (26.3)	203 (26.9)	426 (22.8)
Vascular disease, *n* (%)					
Vascular disease^f^	221 (44.8)	149 (61.3)	226 (60.1)	411 (54.4)	1007 (53.9)
Stroke	37 (7.5)	26 (10.7)	61 (16.2)	91 (12.0)	215 (11.6)
Follow‐up, median [IQR], years	3.0 [1.0, 5.0]	2.0 [0.0, 4.0]	3.0 [1.0, 5.0]	3.0 [1.5, 5.0]	3.0 [1.0, 5.0]

*Note*: Missing data per cohort (Leiden 85‐plus Study = L85+, LiLACS NZ—Māori = NZ‐M, LiLACS NZ—non‐Māori = NZ‐NM, Newcastle 85+ Study = N85+): age—N85+ 1; education—L85+ 3, NZM 1, NZ‐NM 6, N85+ 1; institutionalized—L85+ 30, NZM 4, NZ‐NM 1; chronic diseases—L85+ 13, NZM 2, NZ‐NM 2, N85+ 10; MMSE—L85+ 23, NZM 2, NZ‐NM 12; stroke—NZM 5, NZ‐NM 2.

Abbreviations: IQR, interquartile range; LiLACS NZ, Life and Living in Advanced Age: a Cohort Study in New Zealand; MMSE, Mini‐Mental State Exam; UK, United Kingdom.

^a^ Participants were regarded as institutionalized if living in care‐ or nursing homes in the Leiden 85‐plus Study, in rest homes or private hospitals in LiLACS NZ and in residential care homes, nursing homes or long stay hospitals in the Newcastle 85+ Study.

^b^ Chronic disease was defined as the presence of arthritis, cancer, chronic lung disease, diabetes mellitus or Parkinson's disease.

^c^ Depressive symptoms were defined as a score of ≥2 on the GDS‐12D, the depression subdimension of the GDS.

^d^ Apathy symptoms were defined as a score of ≥2 on the GDS‐3A, the apathy subdimension of the GDS.

^e^ Isolated apathy was defined as having apathy symptoms in the absence of above depressive symptoms (i.e. GDS‐3 A ≥ 2 and GDS‐12D < 2).

^f^ Vascular disease was defined as a history of any clinical atherosclerotic pathology (including angina pectoris, myocardial infarction, intermittent claudication, transient ischemic attack, stroke and any related coronary or peripheral artery surgery).

**FIGURE 1:**
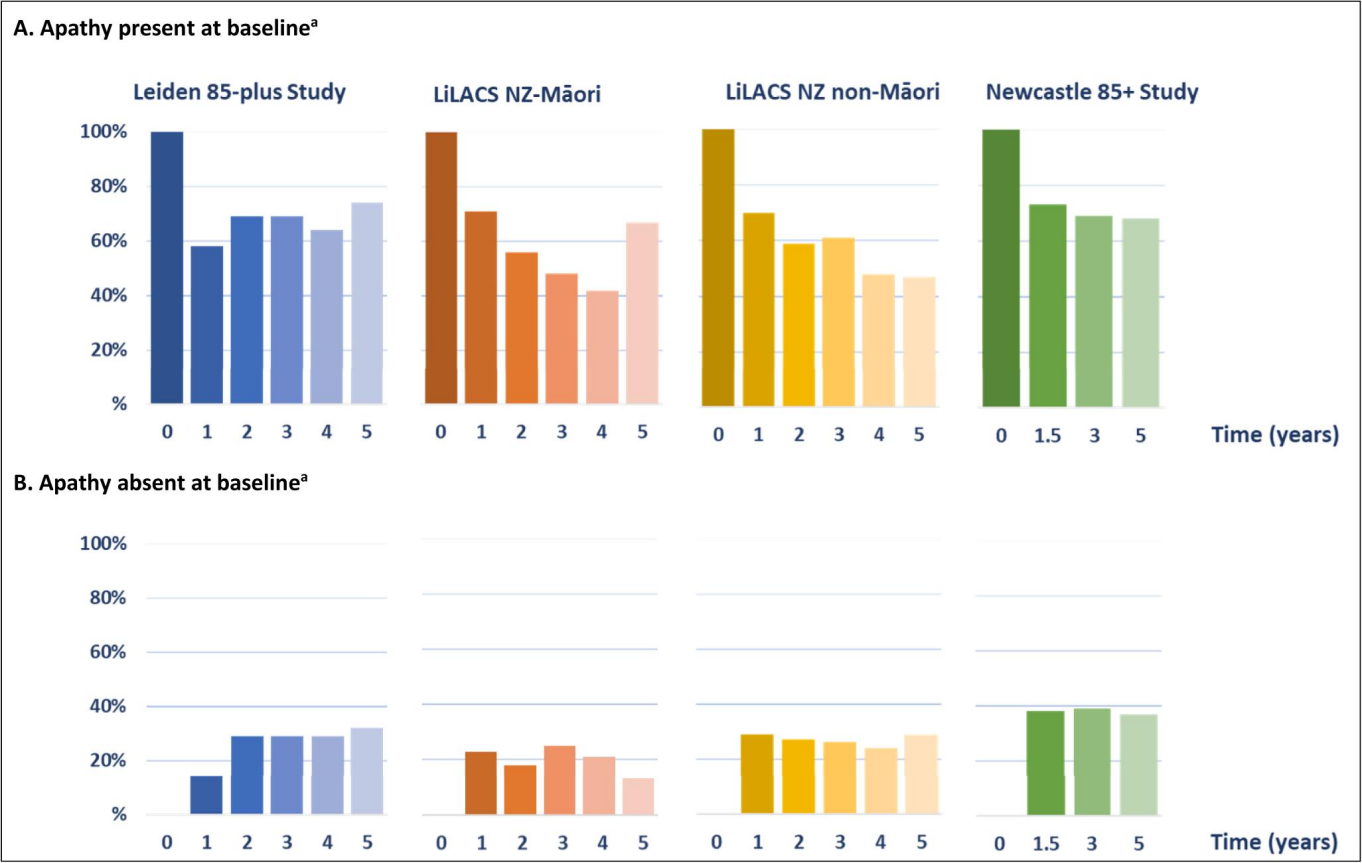
Percentage of participants with apathy symptoms over time, stratified by baseline apathy status. ^a^Baseline apathy status was defined as the presence or absence of apathy symptoms at baseline (i.e. a score of ≥2 or <2 on the GDS‐3A, the apathy subdimension of the GDS). GDS, Geriatric Depression Scale; LiLACS NZ, Life and Living in Advanced Age: a Cohort Study in New Zealand.

Figure 1 shows the percentage of participants experiencing apathy symptoms during follow‐up, stratified by baseline apathy status. The median number of follow‐up waves on the GDS varied from 2 years in the Māori population (IQR 0.0–4.0) to 3 years in the combined cohort (IQR 1.0–5.0). Overall, apathy symptoms occurred at least once again in 65.1% of those experiencing apathy symptoms at baseline, while 42.8% of those without apathy symptoms at baseline experienced apathy symptoms during follow‐up. The majority of both regarded isolated apathy symptoms, respectively 37.3% and 29.9%.

### 3.2 Cross‐sectional and longitudinal associations on vascular apathy

**FIGURE 2:**
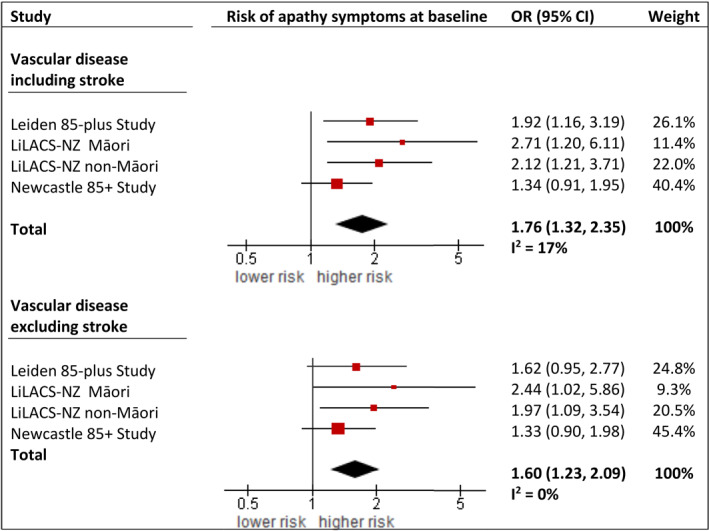
Cross‐sectional association between vascular disease and apathy symptoms. Results from two‐staged, generalized linear mixed model (GLMM) analyses, presented as baseline odds ratios of apathy symptoms (GDS‐3A ≥ 2) by vascular disease status (including and excluding participants with a history of stroke). Fully adjusted models are shown (i.e. adjusted for depressive symptoms, age (only in Māori), sex, education and chronic diseases). Individual study estimates were pooled by random‐effects models with inverse‐variance weighting. CI, confidence interval; GDS, Geriatric Depression Scale; LiLACS NZ, Life and Living in Advanced Age: a Cohort Study in New Zealand; OR, odds ratio.

Adjusted for depressive symptoms, age (only in Māori), sex, education and chronic disease, vascular disease increased the risk of experiencing apathy symptoms at baseline by 76% (pooled OR 1.76, 95% CI 1.32–2.35), as depicted in Figure 2. The association was minimally attenuated and remained significant after exclusion of stroke patients (OR 1.60, 95% CI 1.23–2.09).

**FIGURE 3:**
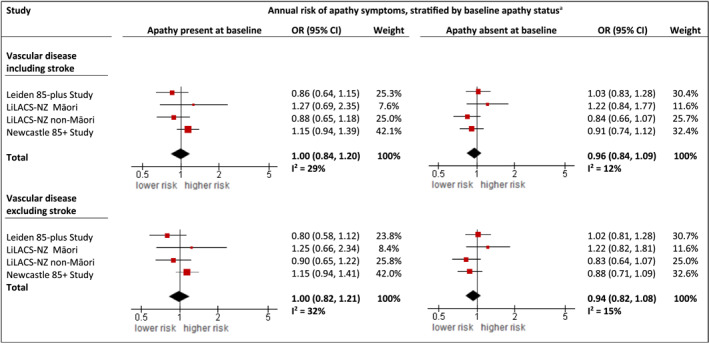
Longitudinal association between vascular disease at baseline and apathy symptoms during follow‐up, stratified by baseline apathy status. Results from two‐staged, generalized linear mixed model (GLMM) analyses, presented as annual odds ratios of apathy symptoms (GDS‐3A ≥ 2) by vascular disease status (i.e. analyses including and excluding participants with a history of stroke). Fully adjusted models are shown (i.e. adjusted for depressive symptoms, age (only in Māori), sex, education and chronic diseases), stratified by baseline apathy status. Individual study estimates were pooled by random‐effects models with inverse‐variance weighting. ^a^Baseline apathy status was defined as the presence or absence of apathy symptoms at baseline (i.e. a score of ≥2 or <2 on the GDS‐3A, the apathy subdimension of the GDS). CI, confidence interval; GDS, Geriatric Depression Scale; LiLACS NZ, Life and Living in Advanced Age: a Cohort Study in New Zealand; OR, odds ratio.

To the contrary, there was no significant association between vascular disease at baseline and the course of apathy symptoms over time, neither in those with apathy symptoms at baseline (OR 1.00, 95% CI 0.84–1.20) nor in those without apathy symptoms at baseline (OR 0.96, 95% CI 0.84–1.09), irrespective of a history of stroke (Figure 3). All minimally adjusted models showed similar results (not shown) and there was overall consistency between the individual estimates (*I* ^2^ < 40%).

### 3.3 Sensitivity analyses

Sensitivity analyses applying the continuous GDS‐3A and GDS‐12D scores showed comparable results for both the cross‐sectional and the longitudinal associations on vascular apathy (not shown).

## 4 DISCUSSION

In this IPD meta‐analysis of four population‐based cohorts of the very old, we found that vascular disease is cross‐sectionally associated with the presence of apathy symptoms, independent of concurrent depressive symptoms and only partially explained by a history of stroke. In contrast, vascular disease was not associated with the occurrence of apathy symptoms over time, which was also irrespective of whether apathy symptoms were experienced at baseline.

In relationship to previous transdiagnostic literature on the vascular apathy hypothesis, our significant cross‐sectional associations are not only consistent in relating vascular disease to apathy in older populations, independent of depression and/or structural brain damage following stroke, but rather add to this body of literature by extending this finding to those of advanced age (i.e. in the very old vs. a mean age of 57 and 74 years old).^13^, ^38^ Our findings were also consistent in direction and magnitude throughout the four cohorts examined suggesting international relevance. This is surprising as the levels of apathetic and depressive symptomatology differed between the cohorts and performance of self‐rated health tools can be culturally or socioeconomically mediated.^39^, ^40^

Conversely, our longitudinal findings in the Leiden 85‐plus Study did not correspond to prior prospective findings by van der Mast et al. on vascular apathy in the same cohort,^11^ presumably due to methodological insights (e.g. different modelling: performing generalized and ordinal regressions on the GDS‐3A instead of linear regressions). In addition, our longitudinal meta‐analysis shows heterogeneity between the cohorts in being more or less likely to display apathy symptoms over time when having vascular disease (not significant). This is not unexpected as NZ Māori have been exposed to a lifetime of discrimination resulting in health disparities,^41^ but suggests nuance in its interpretation. As there are no other prospective, population‐based studies to substantiate the vascular apathy hypothesis,^12^ unequivocal understanding of a potential vascular ground for apathy symptoms remains limited. Optimizing CVD‐management to attempt to prevent apathy in older adults may not be warranted, at least in advanced age.

There could be several pathophysiological mechanisms underlying our findings. One explanation is reversed causality, which has been evidently substantiated in the previously mentioned meta‐analysis.^8^ Apathy symptoms increased the risk of incident myocardial infarction and stroke by 21% and 37% respectively over a median follow‐up of 8.8 years in approximately 48,000 older adults with a median age of 74 years old (IQR 72–78). To elucidate whether our cross‐sectional results reflect this reversed causality or whether our lack of longitudinal evidence is, for instance, explained by characteristics of the TULIPS‐consortium (such as competing risks at advanced age), future research could assess this reversed association for the very old and study vascular apathy in relatively younger, more stable populations of older adults over time (e.g. over 65 years old). In addition, apathy might be a marker of subclinical (cerebral) small vessel disease which could then result in overt CVD.^8^, ^12^ Support for this mechanism is strengthened by associations between more white matter hyperintensities on brain imaging and higher risk of having apathy,^12^, ^42^ which should also be studied prospectively in the general older population to elucidate the direction of their interplay.^12^ Although we did not find support for vascular disease to predispose older adults to apathy symptoms, if the origin were to be vascular, mediation by cognitive decline could explain our lacking prospective associations as older people with severe cognitive impairment could not partake by design. To that extent, upcoming studies on apathy could be complemented by informant‐rated scales or clinical interviews. Lastly, apathy could also be a marker or consequence of (multi)morbidity instead of specifically having a vascular origin; especially the apathy symptoms we studied, as they have been shown to closely connect to functional disability.^43^ This calls for future apathy research to employ the diagnostic criteria^2^ or instruments specifically designed to assess apathy in otherwise healthy, community‐dwelling samples (e.g. the Apathy‐Motivation Index)^44^ to further unravel apathy as an independent construct.

This meta‐analysis has various strengths. It addresses the need for both transdiagnostic and prospective analyses on (vascular) apathy during substantial follow‐up time. The utilized data includes a globally unique collection of population‐based cohort studies of very old adults, originating from diverse countries and cultures, which was honoured by our two‐stage IPD approach. Participants were solely excluded due to data unavailability and by using this IPD‐method we verified previously presented results,^11^ as well as provided consistency in the data and analyses across cohorts.

Although there was not much statistical heterogeneity between the pooled estimates, clinical heterogeneity between the cohorts is a potential limitation, which existed throughout time (i.e. initiation in different time periods) and at baseline (e.g. ethnicity and living situation). Furthermore, a disadvantage inherent to longitudinal research in the very old is the loss‐to‐follow‐up due to death or ill health.^21^ Subgroup analyses were hampered (e.g. minor vs. major vascular disease) and potential other confounders could not be included (e.g. social factors). The associations studied in the TULIPS‐consortium could be even more unraveled by performing a systematic review to find other comparable cohorts. Another limitation is that our conclusions are confined to apathy symptoms of the three included questions in the GDS‐3A and deficits in self‐awareness, commonly associated with apathy,^7^ could have impeded the reliability of this self‐reported assessment. Despite these characteristics the GDS‐3A has however been deemed appropriate for etiological research purposes, particularly in older populations over time.^11^, ^45^

In conclusion, vascular disease in the very old population is associated with apathy symptoms cross‐sectionally, but there is no evidence of influence on apathy occurrence over time, independent of concomitant depressive symptoms. While these findings query the vascular apathy hypothesis and may discourage prevention of apathy by CVD‐management, they emphasize the international relevance of their interplay in advanced age.

## Supporting information

Supplementary Material 1

## Data Availability

The data that support the findings of this study are available from the corresponding author upon reasonable request.
