# Supplementary material for: Vascular disease and apathy symptoms in the very old: A cross‐sectional and longitudinal meta‐analysis of individual participant data
Source: Int J Geriatr Psychiatry. 2022 Oct 31;37(12):10.1002/gps.5831. doi: 10.1002/gps.5831 (PMC9828503; doi:10.1002/gps.5831)
Supplement: Supplementary file 1 — Supplementary Material 1 [file GPS-37-0-s001.docx]

**SUPPLEMENT**

| **Supplementary Table 1**. Additional information on data collection per study of the TULIPS-consortium. | | | |
| --- | --- | --- | --- |
|  | **Leiden 85-plus Study**^1^ | **LiLACS NZ**^2,3a^ | **Newcastle 85+ Study**^4^ |
| **Vascular disease**^b^ | Physician-reported history (by the GP or the elderly care specialist) based on routine medical records; ECG-data on prior MI (Minnesota Code 1-1 or 1-2 (excluding 1-2-8)). | Self-reported history; screening of GP records (except on AP and TIA) and prior hospital admissions ICD-codes (all except AP) (routine medical records); ECG-data on old MI (unspecified coding). | Screening of GP-records (routine medical records); ECG-data on prior MI (Minnesota Codes commencing 1-1 or 5-1 for definite ischemia and commencing 1-2 or 5-2 for probable ischemia). |
| **Chronic disease**^c^ | Physician-reported history or reported disease-related medication use (by the GP or the elderly care specialist) based on routine medical records; checks of pharmacy registries. Drugs were classified by ATC-coding.^d^ Blood sample: Non-fasting glucose > 11.0 mmol/L. | Self-reported history; screening of GP-records (except on arthritis and Parkinson’s) (routine medical records); self-reported medication use; direct observation of pill boxes during interviews. Drugs were classified by ATC-coding.^d^ Blood sample:  Fasting glucose > 7.0 mmol/L. | Screening of GP-records (routine medical records) for history of disease or related medication use. Drugs were classified by BNF-coding.^e^ Blood sample:  Fasting glucose > 7.0 or non-fasting glucose > 11.0 mmol/L. |
| **Apathy symptoms**^e^ | Apathy subdimension of the GDS (i.e. GDS-3A); omitted if a concurrent MMSE-score < 19). The GDS was gathered at baseline and at 1, 2, 3, 4 and 5 years of follow-up. | Apathy subdimension of the GDS (i.e. GDS-3A); excluded for this analysis if a concurrent MMSE-score < 19. The GDS was gathered at baseline and at 1, 2, 3, 4 and 5 years of follow-up. | Apathy subdimension of the GDS (i.e. GDS-3A); omitted if a concurrent MMSE score < 15 and excluded for this analysis if a concurrent MMSE-score < 19. The GDS was gathered at baseline and at 1.5, 3 and 5 years of follow-up. |
| **Notes:** ^a^Data collection for LiLACS NZ was similar for the Māori and non-Māori subpopulations. ^b^Vascular disease was defined as a history of any clinical atherosclerotic pathology (including angina pectoris, myocardial infarction, intermittent claudication, transient ischemic attack, stroke and any related coronary or peripheral artery surgery). ^c^Chronic disease was defined as the presence of arthritis, cancer, diabetes mellitus, chronic lung disease and/or Parkinson’s disease ^d^Utilized ATC-codes: obstructive airway diseases (R03), diabetes (A10) and Parkinson’s (N04A and N04B). ^e^Utilized BNF-codes: obstructive airway diseases (030111, 030120, 030130, 030140 030200, 030210, 030310, 030320), diabetes (060110, 060111, 060112, 060113, 060121, 060122, 060123) and Parkinson’s (040910). ^e^Apathy symptoms were defined as a score of ≥ 2 on the GDS-3A, the apathy subdimension of the GDS.  **Number of missing data** **within each study sample** (Leiden 85-plus Study = L85+, LiLACS NZ – Māori = NZ-M, LiLACS NZ – non-Māori = NZ-NM, Newcastle 85+ Study = N85+): ECG – L85+ 23, NZ-M 27, NZ-NM 38 and N85+ 22. See notes of **Table1** for specifications on any other missing data.  **Abbreviations**: TULIPS, Towards Understanding Longitudinal International older People Studies; LiLACS NZ, Life and Living in Advanced Age: a Cohort Study in New Zealand; GP, general practitioner; ECG, electrocardiogram; MI, myocardial infarction; AP, angina pectoris; TIA, transient ischemic attack; ICD, International Classification of Diseases; ATC, Anatomical Therapeutic Chemical Classification System; BNF, British National Formulary; GDS, Geriatric Depression Scale; MMSE, Mini-Mental State Exam. | | | |

| **Supplementary table 2.** Basic GLMM and approach taken for model selection |
| --- |
| 1. Basic GLMM: glmer(apathy symptoms ~ vascular disease + time + atherosclerosis*time)^a^ 2. Covariates were added based on existing literature,^1,5^ resulting in two models:   **Model 1** adjusted for age (only in Māori), sex and depressive symptoms (as a time-varying covariate) and **Model 2** additionally adjusting for educational attainment and chronic disease.   1. A random intercept was added to both of these models to account for within-participant correlation over time through repeated measures. 2. The addition of a random slope for more detailed between-participant variation in the risk of apathy over time was considered^b^, but it did not provide a significantly better model fit. 3. Stratification: separate models were fit in those with and without baseline apathy to study variation in the risk of experiencing apathy symptoms over time by baseline apathy status. |
| **Notes**: ^a^Generated by use of the glmer-function of the Lme4 R-package. ^b^Generated by use of the mixed_model function of the GLMMadaptive R-package, which allows multiple random effects. **Abbreviations**: GLMM, generalized linear mixed model. |

**REFERENCES – SUPPLEMENT:**

1. van der Mast RC, Vinkers DJ, Stek ML, et al.; Vascular disease and apathy in old age. The Leiden 85-Plus Study. *Int J Geriatr Psychiatry* 2008;**23**(3):266-71. doi: 10.1002/gps.1872.

2. Hayman KJ, Kerse N, Dyall L, et al.; Life and living in advanced age: a cohort study in New Zealand--e Puāwaitanga o Nga Tapuwae Kia Ora Tonu, LiLACS NZ: study protocol. *BMC Geriatr* 2012;**12**:33. doi: 10.1186/1471-2318-12-33.

3. Teh R, Doughty R, Connolly M, et al.; Agreement between self-reports and medical records of cardiovascular disease in octogenarians. *J Clin Epidemiol* 2013;**66**(10):1135-43. doi: 10.1016/j.jclinepi.2013.05.001.

4. Collerton J, Davies K, Jagger C, et al.; Health and disease in 85 year olds: baseline findings from the Newcastle 85+ cohort study. *Bmj* 2009;**339**:b4904. doi: 10.1136/bmj.b4904.

5. Ligthart SA, Richard E, Fransen NL, et al.; Association of vascular factors with apathy in community-dwelling elderly individuals. *Arch Gen Psychiatry* 2012;**69**(6):636-42. doi: 10.1001/archgenpsychiatry.2011.1858.
